# Microvascular Density Analysis of Patients with Inactive Systemic Lupus Erythematosus—A Two-Year Follow-Up Optical Coherence Tomography Angiography Study

**DOI:** 10.3390/jcm13102979

**Published:** 2024-05-18

**Authors:** Martin Dominik Leclaire, Eliane Luisa Esser, Sebastian Dierse, Raphael Koch, Julian Alexander Zimmermann, Jens Julian Storp, Marie-Louise Gunnemann, Larissa Lahme, Nicole Eter, Nataša Mihailovic

**Affiliations:** 1 Department of Ophthalmology, University of Muenster Medical Center, 48149 Muenster, Germany; 2 Institute of Biostatistics and Clinical Research, University of Muenster, 48149 Muenster, Germany; 3 Department of Ophthalmology, Klinikum Bielefeld Gem. GmbH, 33604 Bielefeld, Germany

**Keywords:** SLE, HCQ, vessel density, choriocapillaris, OCT-A, FAZ

## Abstract

**Background/Objectives:** This study aims to investigate the long-term effect of inactive systemic lupus erythematosus (SLE) on the retinal microcirculation measured via optical coherence tomography angiography (OCT-A). **Methods:** Twenty-four eyes of 24 patients with inactive SLE under hydroxychloroquine (HCQ) therapy were included. The OCT-A data (mainly vessel density (VD) and foveal avascular zone (FAZ) data of the superficial and of the deep capillary plexus (SCP, DCP) and the choriocapillaris (CC)) were analyzed and compared between the baseline examination (t_0_) and 2 years later (t_1_). **Results:** At t_1_, VD in the whole en face SCP and in the CC was notably reduced compared to t_0_ (SCP: *p* = 0.001, CC: *p* = 0.013). VD in the DCP, CRT and FAZ area showed no difference at t_1_ compared to t_0_ (DCP: *p* = 0.128, FAZ: *p* = 0.332, CRT fovea: *p* = 0.296). Correlation analysis between the increase in cumulative doses of HCQ between t_0_ and t_1_ and the VD of the whole en face SCP did not show any correlation (Spearman r = 0.062 (95% CI −0.367; 0.477). **Conclusions:** SLE patients demonstrated a decrease in the retinal VD of the SCP and CC over a 2-year period. There was no correlation with the change in cumulative doses of HCQ. These results suggest an ongoing effect of the disease on the retinal and choriocapillary microcirculation.

## 1. Introduction

Systemic lupus erythematosus (SLE) is a multisystemic inflammatory autoimmune disease of the connective tissue. SLE patients suffer from significant morbidity and carry a high mortality risk due to multiple organ and tissue damage [1]. Due to the severe consequences of the disease, research providing new insights into vascular changes in SLE is important, and new insights into SLE pathophysiology and disease activity are desirable. SLE is a chronic disease for which the course often involves years to decades, and accordingly, it usually requires long-term immunotherapy for which hydroxychloroquine (HCQ) represents one of the most common first-line treatment agents [2].

Vascular and microvascular changes, including occlusive vasculopathy, are an important feature of SLE and contribute considerably to the morbidity and mortality in these patients. Vascular alterations in SLE can impair renal function and increase the risk of cardiovascular diseases [3]. Ocular vascular affection in SLE may manifest as optic neuritis, ischemic optic neuropathy, retinal vasculitis, occlusive retinopathies and choroidopathies [4]. Involvement of the posterior segment of the eye usually comes along with poor visual outcomes and is indicative of high disease activity [5].

HCQ was originally an antimalarial drug, and it has been used in the treatment of autoimmune, infectious, metabolic and neoplastic disorders [6]. HCQ decreases disease activity in SLE and improves survival rates [7,8,9,10,11]. HCQ retinopathy is one of its most serious side effects, which might lead to a characteristic bull’s eye maculopathy causing severe and irreversible visual impairment [12]. However, the risk of developing retinopathy is low with a duration of HCQ therapy of less than 5 years, but it increases sharply after more than 5 years of therapy [13].

Optical coherence tomography angiography (OCT-A) is a relatively new imaging technique, and it provides detailed and high-resolution imaging of the retina and choriocapillaris [14,15,16]. OCT-A technology has been described in detail before [14]. Briefly, visualization of the retinal and choriocapillary blood vessels is achieved through the detection of signal differences caused by the movement of blood cells in the otherwise static tissue through multiple, high-resolution scans of a specific area [14,17,18]. In contrast to conventional dye-based methods, such as fluorescein angiography (FLA) and indocyanine green angiography (ICG), OCT-A enables the non-invasive three-dimensional imaging of the retinal and choriocapillary vasculature [19]. Thus, the superficial capillary plexus (SCP) and the deep capillary plexus (DCP) of the retina and the choriocapillaris can be imaged and quantified separately [15,20]. With OCT-A, even minor vascular alterations can be visualized and monitored [21,22]. In contrast to dye-based methods, like FLA and ICG, OCT-A can be performed in cases of dye intolerance, severe renal insufficiency, or pregnancy [14,23]. OCT-A enables the precise visualization and quantification of retinal perfusion and of the foveal avascular zone (FAZ) with good intra-device and intra-personal reproducibility [14,15,16]. Using appropriate software, the automated quantification of the retinal microcirculation is possible. The most used parameters in scientific and clinical applications are the vessel densities (VDs) of the different plexuses and the size of the FAZ. As with conventional OCT images, the retinal thickness can also be measured precisely [17,24].

In a previous publication of our study group, we demonstrated that SLE has a negative impact on the retinal vessel density (VD) of the superficial capillary plexus (SCP) measured via OCT-A despite a clinically inactive disease status. At the same time, we could not demonstrate any effects of HCQ on retinal VD, apart from a positive correlation of cumulative HCQ doses in a group of patients with a short duration of HCQ therapy, neither by HCQ dose nor by the duration of intake. On the contrary, our results suggested that HCQ might even have a protective effect on the retinal microvasculature. Moreover, choriocapillary (CC) VD and the area of the foveal avascular zone (FAZ) were significantly impaired [25]. An impaired retinal VD in SLE patients has also been described in other studies [26,27,28].

The aim of this study was to compare the VD in SLE patients treated with HCQ after a period of two years thus investigating long-term effects of the clinically apparent inactive disease on the retinal microvasculature.

Therefore, we aimed to investigate retinal whole en face VD in the SCP in SLE patients over the course of two years and to identify a possible relationship between VD changes over time and cumulative HCQ doses.

Further, the quantitative analysis of the retinal VD in the deep capillary plexus (DCP) and CC, the central retinal thickness (CRT) and FAZ area were explored. These measurements were analyzed in more detail in the two subgroups. The subgroups were defined as a low-risk group (therapy duration less than 5 years at baseline investigation (t_0_)) and a high-risk group (therapy duration of more than or equal to 5 years at t_0_) for HCQ-induced retinopathy. The differences between t_0_ and t_1_ in the subgroups were also compared.

## 2. Materials and Methods

This exploratory observational study investigated 24 eyes of 24 patients with SLE over a period of two years. The study was approved by the Ethics Committee of Westphalia-Lippe, Germany (No.: 2016324-f-S). Before performing any examination, the study protocol was explained in detail and all participants signed an informed consent form. The study adhered to the tenets of the Declaration of Helsinki.

### 2.1. Patient Inclusion Criteria

Detailed patient records on SLE disease progression and concomitant diseases (systemic and ophthalmologic) were available for all included patients. Patients with concomitant diseases that could influence the OCT-A parameters were excluded, particularly patients with arterial hypertension, diabetes mellitus, nicotine abuse and other autoimmune diseases other than SLE, such as rheumatoid arthritis, as well as relevant opacities of the optical media, glaucoma, high myopia or retinal diseases.

Only patients with inactive SLE were included. Five patients had an episode of lupus nephritis occurring before the study period. None of the patients received treatment for active nephritis during the study. Asymptomatic mitral valve insufficiency was described in the records of 2 patients. The mitral valve insufficiency did not occur during the study period and neither of the two patients required mitral valve surgery.

### 2.2. Examinations

All study participants underwent an ophthalmic examination, including an anterior segment examination, binocular fundus examination and OCT-A imaging. These examinations were performed at baseline (t_0_) and after two years (t_1_).

In the patient group, 10–2 visual field testing (standard automated perimetry), SD-OCT using the Spectralis OCT (Heidelberg Engineering, Heidelberg, Germany), fundus autofluorescence and multifocal electroretinograms were performed to rule out HCQ-associated retinopathy. Only patients with no signs of HCQ toxicity were included in this study.

### 2.3. Definition of Subgroups According to Duration of HCQ Intake

Patients treated with HCQ for >5 years at t_0_ were classified as the “high-risk group” (*n* = 12); patients with a HCQ treatment for ≤5 years at baseline were classified as the “low-risk group” (*n* = 12). This classification was based on the revised recommendations on screening for HCQ therapy of the American Academy of Ophthalmology on screening for chloroquine and HCQ retinopathy, which define a duration of use >5 years as a factor of increasing the risk of HCQ-retinopathy. For the correlation analysis of the VD and the cumulative dose of HCQ, cumulative dose data could be extracted from all patient records (*n* = 24).

### 2.4. Optical Coherence Tomography Angiography

OCT-A imaging of all subjects was performed with the AngioVue™ Imaging System (RTVue XR Avanti with AngioVue; Optovue Inc., Fremont, CA, USA). The split-spectrum amplitude-decorrelation angiography algorithm is used to create OCT-A data. VD measurements were created automatically and displayed and analyzed using Revue software (version 2017.1.0.151, Optovue Inc., Fremont, CA, USA). The OCT-A technology has been described elsewhere in detail [21]. OCT-A imaging of the macula was performed using a 3 × 3 mm scan. Only OCT-A images of good quality (quality index ≥ 6) were included, and images with lines or gaps due to poor signal strength or motion artefacts were excluded from the study [22]. The automated segmentation was checked by an experienced reader before data analysis.

### 2.5. Statistics

Data were collected in Microsoft Excel 2016. Statistical analyses were performed using IBM SPSS Statistics for Windows, Version 29 (IBM, Armonk, NY, USA). Continuous variables are reported as the median (25% quantile; 75% quantile, interquartile range). Pairwise comparisons of baseline and 2-year values were performed using two-sided exact Wilcoxon signed-rank tests. Boxplots were used for the graphical presentation. The patient-wise differences between t_1_ and to are denoted as Δ and were compared between the two subgroups (high-risk and low-risk) using two-sided exact Mann–Whitney U tests. Correlations between two continuous variables are reported as Spearman’s correlation coefficient (r) and 95% bias-corrected and accelerated (BCa) bootstrap confidence intervals (CI) using 50.000 samples. All *p*-values and confidence limits were two-sided and intended to be exploratory rather than confirmatory. Therefore, no adjustment for multiplicity was made. Exploratory two-sided *p*-values ≤ 0.05 were considered statistically noticeable.

## 3. Results

The same collective was examined at t_0_ and t_1_. The demographic characteristics of the study population and of the high/low-risk subgroups are shown in Table 1.

Statistical analysis revealed a noticeable reduction in the VD in the whole en face SCP of the whole study group when comparing the examinations at t1 to t_0_ (Δt_1_ − t_0_, abbreviated as Δ in the following) (Δ whole en face SCP: −1.4 (interquartile range (IQR) −3.3; −0.1), *p* = 0.001).

Moreover, VD in the parafoveal region of the SCP and of the CC angiogram were reduced at t_1_ compared to t_0_ (Δ parafoveal SCP: −1.5 (IQR −3.9; −0.2), *p* < 0.001; Δ parafoveal CC: −0.9 (IQR −2.6; 0.4), *p* = 0.013)). There was no noticeable change in the VD in the SCP of the foveal region (Δ: −0.6 (IQR 2.8; −0.9), *p* = 0.241) or in the VD in the DCP (Δ whole en face DCP: −1.3 (IQR −3.1; 0.7), *p* = 0.128, Δ fovea DCP: 0.1 (IQR −1.0; 1.3), *p* = 0.671, Δ parafovea DCP: −1.4 (IQR −2.8; 0.5), *p* = 0.074), the CRT (Δ fovea CRT: 0.6 (IQR −1.4; 2.6), *p* = 0.269) or FAZ area (Δ: 0.01 (IQR −0.01; 0.03), *p* = 0.332) (Table 2).

**Figure 1:**
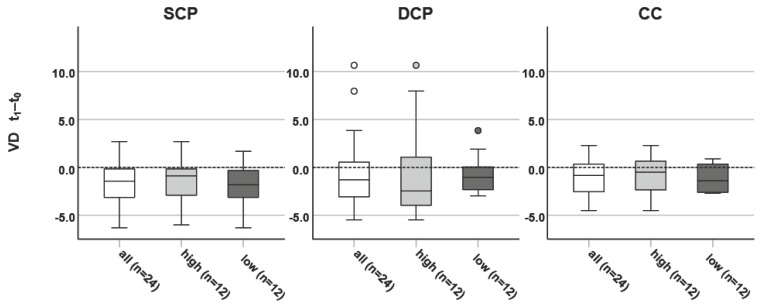
Boxplots of differences between the examination after two years (t_1_) and baseline (t_0_) for the vessel densities (VDs) of (**left**) the superficial retinal capillary plexus (SCP) of the whole en face (WEF) optical coherence tomography angiogram (OCT-A). (**middle**) VD of the deep retinal capillary plexus (DCP) WEF OCT-A (**right**) of the choriocapillary (CC) WEF VD. The boxplot shows the quartiles as a box, “whiskers”—denoting the most extreme data point that is no more than 1.5 times the IQR from the central box—and more extreme data points, which are plotted individually.

The Δ of the whole en face SCP, DCP and CC VD are also displayed as box plot diagrams in Figure 1.

**Figure 2:**
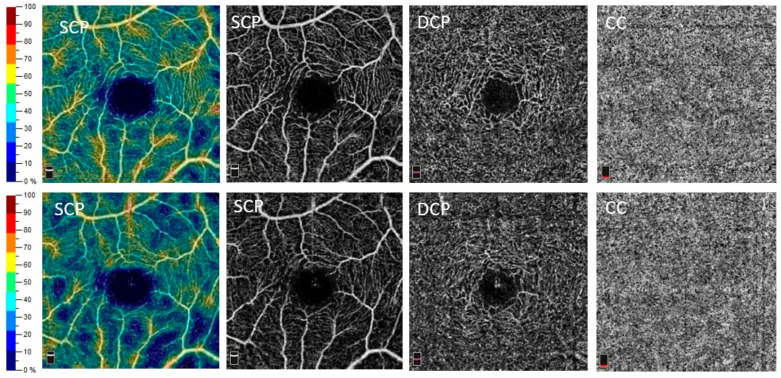
Exemplary images of the optical coherence tomography angiography (OCT-A) vessel density (VD) measurements. These are measurements from the same individual at baseline (t_0_, (**top**)) and after two years (t_1_, (**bottom**)). Displayed are from left to right: heat map of the VD of the superficial capillary plexus (SCP), vessel visualization of the SCP, vessel visualization of the deep capillary plexus (DCP) and the choriocapillaris (CC). Note the reduced VD in the SCP heat map at t_1_ compared to t_0_.

The reduction in VD between t0 and t1 was not only measurable but also visible in some eyes in the OCT angiogram or in the heat map of the OCT angiogram, as shown in Figure 2.

When analyzing the data of the subgroup “high-risk patients”, there was a trend of a reduction in the VD at t1 compared to t0 in the whole en face SCP (Δ: −0.9 (IQR −3.2; −0.1), *p* = 0.052) (Table 3).

A comparison of the Δ of VD data of the subgroup “low-risk patients” revealed a reduced VD in the whole en face SCP (Δ: −1.8 (IQR −3.3; −0.1), *p* = 0.012) and the parafoveal SCP (Δ: −2.1 (IQR 4.0; −0.4), *p* = 0.009) (Table 4).

No noticeable differences were observed when comparing the Δ in VD, CRT, and FAZ size between the high-risk and the low-risk groups. (Table 5).

Correlation analysis of cumulative HCQ dosage increase and CC change in VD demonstrated a negative correlation (r = −0.627) within the low-risk group, implying that a dosage increase since baseline was linked to a decline in CC perfusion. No monotone correlation was found between the cumulative increase in HCQ dose since baseline and the change in SCP or DCP VD, neither in the whole study group nor in the high-/low-risk subgroups (all Spearman’s correlation coefficients −0.238 ≤ r ≤ 0.301) (Supplemental Table S1).

## 4. Discussion

Optical coherence tomography angiography is a relatively new technique, which offers a uniquely simple, non-invasive visualization of the microcirculation [29]. The automated VD measurements using the Revue software enable measurements with good repeatability and reproducibility [30,31,32,33]. Vascular disease is frequent in patients with SLE, and it is the major cause of death-established SLE. Vascular disease in SLE can manifest as accelerated atherosclerosis predisposing to coronary artery disease and renal disease, which are major causes of mortality [34,35]. HCQ therapy is the standard treatment for SLE, which can lead to HCQ-induced maculopathy as one of the most serious complications. Since HCQ is widely used in the treatment of SLE as it reduces SLE-associated mortality, a deeper understanding of HCQ retinopathy, the pathophysiology of which remains incompletely understood, is highly desirable [6].

Regarding the effect of SLE on retinal VD in patients with HCQ treatment, the results of this study are in line with the findings of one of our previous studies [25]. In SLE patients, the disease itself and not the HCQ therapy seems to be the main cause for the retinal VD reduction, as there was no correlation between the dosage increase since baseline of HCQ and the SCP and DCP VD. The decrease in VD after two years suggests that SLE leads to a continuous alteration in retinal blood flow regardless of absent clinical disease activity. Interestingly, a negative correlation could be observed in the low-risk group between CC VD and the increase in the cumulative dose of HCQ. In a previous study, we demonstrated a positive correlation between the total dose of HCQ and retinal VD in a group with a short duration of HCQ intake (less than 5 years), and we postulated a possible protective effect of HCQ on retinal perfusion in the early phase of therapy [25]. It is possible that HCQ affects the CC microcirculation differently from the retinal microcirculation, suggesting complex and opposing effects of HCQ on the retinal and choriocapillary microcirculation.

In contrast to our results, previous studies have postulated a significant effect of HCQ intake on retinal VD in patients with various underlying diseases [36]. However, the results of these studies do not allow conclusions to be drawn as to whether the measured VD reduction actually results from HCQ therapy or the underlying disease. In this context, it is worth mentioning that there are also other studies clearly lacking evidence that HCQ has an influence on vascular dysfunction. In a previous study involving patients with rheumatoid arthritis and HCQ therapy, no difference in VD was observed between the patient group and the control group. Similarly, there was no correlation found between the cumulative HCQ dosage or duration of HCQ intake and VD [37]. This study, including a correlation analysis, uniquely comprised patients solely with rheumatoid arthritis and HCQ intake, excluding other autoimmune conditions where the disease itself could affect the microvasculature. Thus far, from our perspective, it appears that one cannot conclusively assume that HCQ has a definite impact on VD.

In a recent meta-analysis, Ferreira et al. examined the effect of HCQ therapy on retinal perfusion [36]. In this analysis, it is postulated that HCQ therapy is associated with a significantly decreased retinal VD. It is assumed that the measured VD reduction is due to HCQ therapy, but this cannot ultimately be concluded against the background of the underlying diseases of the patients. Furthermore, this meta-analysis included studies that examined either patients with SLE or patients with rheumatoid arthritis, respectively. For rheumatoid arthritis, we could not demonstrate any influence of the disease on VD in OCT-A as stated in a previous publication [37]. This underlines that patients with different underlying diseases using HCQ should not be grouped when investigating the influence of HCQ on VD, as this could significantly bias the results.

The progressive reduction in retinal VD over two years suggests that SLE itself, as a chronic connective tissue disease, leads to the continuous deterioration of retinal perfusion even when the disease is clinically apparently inactive. Vascular changes in SLE patients can lead to renal function impairment and cardiovascular disease (CVD), increasing mortality [38,39]. However, these changes are not easy to detect and monitor, especially if they are subtle and sub-clinical. In contrast to OCT-A, the examination techniques used for detecting and monitoring these changes are mostly invasive, such as kidney biopsies, or require experience and training, such as ultrasound exams [40,41]. Vascular alterations leading to CVD are one of the leading causes of morbidity and mortality in SLE patients, and evidence indicates that clinical risk scores, which were designed for the general population, underestimate the risk of CVD in SLE patients [42,43,44]. The reasons for the progressive VD reduction are speculative. Small vessel vasculitis is a common manifestation of SLE affecting around 50% of SLE patients [45]. Antibodies against endothelial cells have been hypothesized to be implicated in its pathogenesis [46]. One could hypothesize that patients with inactive SLE might have a subclinical form of vasculitis, but further research is needed to clarify the mechanisms that lead to the microvascular alterations.

Our results suggest that OCT-A might be useful to monitor disease progression or the vascular status in SLE patients. The simple and rapid feasibility, as well as the non-invasiveness of OCT-A, would be an advantage over other, possibly more invasive examination methods. However, based on the current state of knowledge, no concrete recommendations can yet be made on the use of this technology in SLE patients. In addition, even though OCT-A measurements are available at more and more centers, they are not yet widely used in routine clinical practice. Further studies investigating the correlation and long-term effects between the OCT-A parameters and other established markers, such as renal function or cardiac output, would be highly desirable.

The retina is supplied with blood from two distinct vascular systems, the retinal blood vessels and the choroid. The retinal blood vessels are derived from the central retinal artery, whereas the choroid is derived from the ciliary arteries [47]. We demonstrated in a previous study that the CC VD is reduced in SLE patients compared to healthy controls [25]. Our results in this present study show that VD also decreases noticeably in the choriocapillaris region over the period of 2 years, regardless how long the patients are on HCQ therapy. The choroidal and the retinal vasculature differ in terms of blood flow, autoregulation and oxygen saturation of their blood [48,49]. The fact that SLE affects the flow density of two distinguished vascular plexuses in the eye and further reduces the VD during the course of the disease suggests systemic effects on the microcirculation, which has been demonstrated in previous studies using different techniques, such as nailfold capillaroscopy or combined laser Doppler flowmetry/diffuse reflectance spectroscopy [50,51].

Over the course of two years, this study showed no noticeable difference regarding CRT. In a prior publication with a mostly identical group of patients, we found no difference in CRT compared to a healthy control group. The sensitivity of the RT analysis in detecting HCQ retinopathy has been demonstrated previously [52,53,54]. The absence of CRT deterioration during disease progression in SLE patients argues against an HCQ-induced retinopathy in our patient group. This supports the hypothesis that SLE is the main reason for the decreased VD and not HCQ.

Limitations of this study are that the included patients have different histories of SLE and that the baseline examination was not performed at the time of the diagnosis of the disease, which were instead performed in different patients at different time points during HCQ treatment. However, the study group was still quite homogenous since we ruled out HCQ-related retinopathy, and none of the patients had clinically active disease. An investigation of VD dynamics starting with the initial diagnosis of the disease in another prospective study would be desirable. A limitation of many OCT-A-based studies is the fact that, to date, there are no generally recognized age-, ethnicity- and sex-correlated norm values, so the measured differences are relative. It is known that the intrapersonal reproducibility of the OCT-A measured values (both with regard to VD and FAZ) is very good [31,32,55], so that valid differences between t_1_ and t_0_ can be assumed, which were also statistically noticeable. Another limitation is the relatively small size of the study population (*n* = 24), which is due to the relative rarity of the disease and the strict exclusion criteria that had to be formulated for the validity of the OCT-A measurements. The long study duration of two years also limited the number of study participants, as valid and high-quality measurements had to be available over this period. In view of the rarity of the disease, the exclusion criteria and the long observation period, a study population of 24 is nevertheless a considerable number and allows for valid conclusions to be drawn. To the best of our knowledge, this is the only study that has examined the OCT-A parameters in SLE patients over a long observation period. In a previous study, we compared OCT-A parameters between SLE patients and healthy controls [25]. Since, in contrast to the SLE patients, the healthy participants were not followed over the study period of two years, this study does not have a control group, which is another limitation. However, good long-term repeatability and reproducibility of OCT-A measurements in healthy subjects have been reported [33]. It can therefore be concluded that, despite the lack of a healthy control group, the measured VD reduction in SLE patients is valid and cannot be attributed to a mere time- and age-related reduction.

There were differing results regarding SCP and DCP. It is known that the fluctuation range of the DCP values is greater [56,57], so that less accurate measurements can be assumed here, which may make the results less valid and therefore, differences may not be displayed in full extend.

## 5. Conclusions

In summary, this study observes for the first time that SLE reduces the retinal microcirculation over time, even when the patients are under treatment with HCQ and have clinically inactive disease without signs of HCQ-related retinopathy. The lack of a correlation between the change in retinal VD and the increase in the cumulative HCQ dose or duration of therapy argues against an effect of HCQ on VD measurements. OCT-A could be a promising imaging modality for disease monitoring in SLE patients.

## Figures and Tables

**Table 1:** Demographic data of the study population.

	Total Study Group	High-Risk Group	Low-Risk Group
n	24	12	12
Sex f/m	14/10	5/7	9/3
Age (years) at t_0_	47.0 (34.0; 54.8)	43.0 (32.5; 54.8)	48.0 (37.5; 54.3)
Duration HCQ therapy (years) at t_0_	5.1 (2.5; 13.2)	12.0 (6.1; 15.8)	2.5 (1.3; 3.8)
Duration HCQ therapy (years) at t_1_	7.2 (4.4; 15.0)	13.7 (7.9; 17.3)	4.4 (3.4; 6,2)
Cumulative dose HCQ (g) at t_0_	532.8 (317.6; 1313.6)	1223.9 (466.9; 1640.8)	322.7 (164.8; 538.2)
Cumulative dose HCQ (g) at t_1_	856.2 (516.3; 1583.9)	1518.2 (746.2; 1937.9)	587.7 (398.2; 858.6)

f = female, g = gram, HCQ = hydroxychloroquine, m = male, t_0_ = baseline, t_1_ = two years after baseline. Low-risk-group: systemic lupus erythematosus (SLE) patients treated with HCQ ≤ 5 years. High-risk-group: SLE patients treated with HCQ > 5 years. Continuous variables are reported as the median (25% quantile; 75% quantile). Note: in some patients, HCQ therapy was paused temporarily or HCQ doses were adjusted. Consequently, the values between t_0_ and t_1_ did not simply sum up.

**Table 2:** Vessel density (VD,%) data of the SLE group at baseline (t_0_) and after two years (t_1_).

Total Study Group				
	Baseline (t_0_) (*n* = 24)	2 Years (t_1_) (*n* = 24)	Δt_1_ − t_0_ (*n* = 24)	*p*
SCP (VD)				
whole en face	47.0 (45.5; 47.8)	45.3 (43.4; 46.9)	−1.4 (−3.3; −0.1)	**0.001**
fovea	20.3 (15.5; 23.3)	18.4 (13.6; 23.3)	−0.6 (−2.8; −0.9)	0.241
parafoveal	50.0 (47.6; 50.5)	47.9 (45.4; 49.8)	−1.5 (−3.9; −0.2)	**<0.001**
DCP (VD)				
whole en face	51.1 (48.9; 51.8)	49.9 (46.6; 51.9)	−1.3 (−3.1; 0.7)	0.128
fovea	37.2 (29.2; 40.9) *	35.7 (29.6; 41.6)	0.1 (−1.0; 1.3) *	0.671
parafoveal	53.4 (51.2; 54.0)	52.4 (48.4; 54.1)	−1.4 (−2.8; 0.5)	0.074
CC (VD)	70.9 (66.3; 73.6)	69.7 (64.3; 72.6)	−0.9 (−2.6; 0.4)	**0.013**
CRT (µm)				
fovea	260.4 (246.4; 271.1)	261.9 (245.9; 278.2)	0.6 (−1.4; 2.6)	0.296
parafoveal	327.1 (315.1; 335.1)	325.3 (317.7; 334.8)	−0.2 (−1.8; 1.6)	0.961
FAZ (mm^2^)	0.26 (0.19; 0.34)	0.27 (0.23; 0.36) *	0.01 (−0.01; 0.03) *	0.332

CC = choriocapillaris, CRT = central retinal thickness, DCP = deep retinal capillary plexus, SCP = superficial retinal capillary plexus, VD = vessel density, * missing value of one patient. Changes between t_1_ and t_0_ (Δt_1_ − t_0_) obtained in the regions indicated. Continuous variables are reported as the median (25% quantile; 75% quantile). *p*-values are from pairwise comparisons of baseline and two-year values using two-sided exact Wilcoxon signed-rank tests. Bold: *p*-values ≤ 0.05.

**Table 3:** Vessel density (VD,%) data of the high-risk subgroup (hydroxychloroquine therapy duration > 5 years at baseline (t_0_) and after two years (t_1_).

High-Risk Group				
	Baseline (t_0_) (*n* = 12)	2-Years (t_1_) (*n* = 12)	Δt_1_ − t_0_ (*n* = 12)	*p*
SCP (VD)				
whole en face	46.8 (42.7; 47.5)	45.4 (43.5; 47.8)	−0.9 (−3.2; −0.1)	0.052
fovea	19.8 (15.5; 24.2)	17.9 (16.7; 24.2)	−0.4 (−2.5; 0.6)	0.424
parafoveal	49.8 (45.2; 50.4)	47.6 (46.3; 51.2)	−1.3 (−3.7; −0.2)	0.077
DCP (VD)				
whole en face	50.4 (47.2; 51.4)	47.4 (45.9; 52.6)	−2.5 (−4.0; 1.2)	0.470
fovea	37.2 (29.2; 41.9) *	35.7 (29.7; 39.4)	−0.2 (−2.1; 0.7) *	0.577
parafoveal	52.6 (50.0; 54.0)	49.7 (48.1; 54.7)	−1.5 (−4.1; 1.1)	0.301
CC (VD)	71.5 (68.1; 74.8)	72.2 (65.5; 73.5)	−0.5 (−2.4; 0.9)	0.176
CRT (µm)				
fovea	258.2 (249.4; 268.2)	255.5 (246.5; 279.6)	0.6 (−3.1; 2.1)	0.910
parafoveal	334.0 (317.3; 335.8)	331.1 (316.9; 337.2)	−0.8 (−3.2; 2.0)	0.349
FAZ (mm^2^)	0.25 (0.21; 0.34)	0.25 (0.22; 0.31)	0.01 (−0.01; 0.04)	0.758

CC = choriocapillaris, CRT = central retinal thickness, DCP = deep retinal capillary plexus, SCP = superficial retinal capillary plexus, VD = vessel density, * missing value of one patient. Changes between t_1_ and t_0_ (Δt_1_ − t_0_) obtained in the regions indicated. Continuous variables are reported as the median (25% quantile; 75% quantile). *p*-values are from pairwise comparisons of baseline and two-year values using two-sided exact Wilcoxon signed-rank tests. Bold: *p*-values ≤ 0.05.

**Table 4:** Vessel density (VD,%) data of the low-risk subgroup (hydroxychloroquine therapy duration ≤ 5 years) at baseline (t_0_) and after two years (t_1_).

Low-Risk Group				
	Baseline (t_0_) (*n* = 12)	2-Years (t_1_) (*n* = 12)	Δt_1_ − t_0_ (*n* = 12)	*p*
SCP (VD)				
whole en face	47.0 (45.5; 48.1)	45.2 (43.0; 46.3)	−1.8 (−3.3; −0.1)	**0.012**
fovea	20.5 (15.5; 22.9)	18.6 (12.7; 23.3)	−1.2 (−3.5; 1.5)	0.424
parafoveal	50.2 (47.7; 51.1)	47.9 (44.7; 49.5)	−2.1 (−4.0; −0.4)	**0.009**
DCP (VD)				
whole en face	51.4 (49.3; 52.5)	51.1 (49.0; 51.8)	−1.0 (−2.3; 0.2)	0.176
fovea	35.3 (28.9; 40.6)	35.6 (28.2; 42.2)	0.7 (−0.7; 1.6)	0.151
parafoveal	53.6 (52.2; 54.4)	52.9 (51.9; 54.1)	−0.8 (−2.6; 0.3)	0.204
CC (VD)	69.2 (62.9; 72.0)	68.6 (63.0; 70.4)	−1.4 (−2.6; 0.4)	**0.027**
CRT (µm)				
fovea	262.3 (240.8; 278.6)	267.8 (242.3; 278.2)	1.5 (0.2; 3.2)	0.230
parafoveal	321.5 (313.7; 327.9)	321.9 (318.2; 328.2)	0.9 (−1.0; 1.6)	0.370
FAZ (mm^2^)	0.28 (0.14; 0.34)	0.30 (0.27; 0.39) *	0.01 (−0.01; 0.02) *	0.363

CC = choriocapillaris, CRT = retinal thickness, DCP = deep retinal capillary plexus, SCP = superficial retinal capillary plexus, VD = vessel density, * missing value of one patient. Changes between t_1_ and t_0_ (Δt_1_ − t_0_) obtained in the regions indicated. Continuous variables are reported as the median (25% quantile; 75% quantile). *p*-values are from pairwise comparisons of baseline and two-year values using two-sided exact Wilcoxon signed-rank tests. Bold: *p*-values ≤ 0.05.

**Table 5:** Comparison of vessel density (VD,%) between the high-risk group and the low-risk group regarding the differences between baseline and after two years (Δt_1_ − t_0_).

	Δt_1_ − t_0_ High-Risk Group (*n* = 12)	Δt_1_ − t_0_ Low-Risk Group (*n* = 12)	*p*
SCP (VD)			
whole en face	−0.9 (−3.2; −0.1)	−1.8 (−3.3; −0.1)	0.640
fovea	−0.4 (−2.5; 0.6)	−1.2 (−3.5; 1.5)	0.932
parafoveal	−1.3 (−3.7; −0.2)	−2.1 (−4.0; −0.4)	0.443
DCP (VD)			
whole en face	−2.5 (−4.0; 1.2)	−1.0 (−2.3; 0.2)	0.410
fovea	−0.2 (−2.1; 0.7)	0.7 (−0.7; 1.6)	0.211
parafoveal	−1.5 (−4.1; 1.1)	−0.8 (−2.6; 0.3)	0.713
CC (VD)	−0.5 (−2.4; 0.9)	−1.4 (−2.6; 0.4)	0.640
CRT (µm)			
fovea	0.6 (−3.1; 2.1)	1.5 (0.2; 3.2)	0.325
parafoveal	−0.8 (−3.2; 2.0)	0.9 (−1.0; 1.6)	0.272
FAZ (mm^2^)	0.01 (−0.01; 0.04)	0.01 (−0.01; 0.02) *	0.727

CC = choriocapillaris, CRT = central retinal thickness, DCP = deep retinal capillary plexus, SCP = superficial retinal capillary plexus, VD = vessel density, * missing value of one patient. Changes between t_1_ and t_0_ (Δt_1_ − t_0_) obtained in the regions indicated. Continuous variables are reported as the median (25% quantile; 75% quantile). *p*-values are from two-sided exact Mann–Whitney U tests.

## Data Availability

Data are available upon request from the corresponding author.

## Supplementary Materials

The following supporting information can be downloaded at: https://www.mdpi.com/article/10.3390/jcm13102979/s1, Table S1: Correlation analysis for the whole study group, the high-risk group and the low risk group for the differences between two years and baseline (Δ_t1−t0_) for the hydroxychloroquine (HCQ) cumulative doses and Δ_t1−t0_ of the vessel densities in the regions indicated.
