# Supplementary material for: Microvascular Density Analysis of Patients with Inactive Systemic Lupus Erythematosus—A Two-Year Follow-Up Optical Coherence Tomography Angiography Study
Source: J Clin Med. 2024 May 18;13(10):2979. doi: 10.3390/jcm13102979 (PMC11122300; doi:10.3390/jcm13102979)
Supplement: Supplementary file 1 [file jcm-13-02979-s001.zip › jcm-3003241-supplementary.pdf]

**Table S1.** Correlation analysis for the whole study group, the high-risk group and the low risk group for the differences between two years and baseline ($\Delta_{t1-t0}$) for the hydroxychloroquine (HCQ) cumulative doses and $\Delta_{t1-t0}$ of the vessel densities in the regions indicated.

| | | | | |
|---|---|---|---|---|
| **Whole Study Group (n = 24)** | | | | |
| | | $\Delta_{t1-t0}$ VD SCP *whole en-face* | $\Delta_{t1-t0}$ VD DCP *whole en-face* | $\Delta_{t1-t0}$ VD CC |
| $\Delta_{t1-t0}$ cumulative HCQ doses | r (95% CI) | 0.062 (−0.367; 0.477) | 0.246 (−0.143; 0.576) | −0.182 (−0.681; 0.317) |
| **High-Risk Group (n = 12)** | | | | |
| | | $\Delta_{t1-t0}$ VD SCP *whole en-face* | $\Delta_{t1-t0}$ VD DCP *whole en-face* | $\Delta_{t1-t0}$ CC |
| $\Delta_{t1-t0}$ cumulative HCQ doses | r (95% CI) | 0.119 (−0.604; 0.796) | 0.301 (−0.335; 0.706) | 0.182 (−0.590; 0.746) |
| **Low-Risk Group (n = 12)** | | | | |
| | | $\Delta_{t1-t0}$ VD SCP *whole en-face* | $\Delta_{t1-t0}$ VD DCP *whole en-face* | $\Delta_{t1-t0}$ CC |
| $\Delta_{t1-t0}$ cumulative HCQ doses | r (95% CI) | −0.063 (−0.696; 0.602) | −0.238 (−0.810; 0.596) | −0.627 (−0.929; 0.052) |

DCP = deep retinal capillary plexus, HCQ = hydroxychloroquine, SCP = superficial retinal capillary plexus, VD = vessel density, r = Spearmans's correlation coefficient, 95% CI = 95% confidence interval. Displayed are the Spearman's rank correlation coefficients (r) and 95% bias-corrected and accelerated (BCa) bootstrap confidence intervals (CI) using 50.000 samples. High-risk group: HCQ therapy duration > 5 years, low risk group: HCQ therapy duration ≤ 5 years.
